# LINC00488 stimulates the progression of esophageal cancer by targeting microRNA-485-5p

**DOI:** 10.3892/ol.2022.13412

**Published:** 2022-07-04

**Authors:** Hongbo Xu, Yan Ye

Oncol Lett 21: 86, 2021; DOI: 10.3892/ol.2020.12347

Following the publication of this paper, it was drawn to the Editors’ attention by a concerned reader that one of the panels showing the results from cell migration assay experiments in Fig. 4C on p. 7 contained a section of data that were overlapping with data presented in another paper written by other authors and published in a different journal. Furthermore, a problematic issue was also noted with the Kaplan-Meier plots in Fig. 3C on p. 6, where the units along the *x*-axis (time in months) were not easily reconcilable with the dates reported for the collection of the patients’ samples.

Given the issue of the overlapping section of data and the difficulties associated with the presentation of the Kaplan-Meier plots, the Editor of *Oncology Letters* has decided that this paper should be retracted from the Journal on account of the sharing of data and a lack of confidence in the presented results. The authors were asked for an explanation to account for these concerns, but the Editorial Office did not receive a reply. The Editor apologizes to the readership for any inconvenience caused.

